# Dr. Daniel’s New Book*From The American Journal of Clinical Medicine.

**Published:** 1907-03

**Authors:** George H. Candler

**Affiliations:** Chicago, Ill.


### Dr. Daniel's New Book.*

"The Strange Case of Dr. Bruno," by F. E. Daniel, M. D. The Guarantee Pub. Co., New York. Price, $1.50.

Those who have had the pleasure of knowing Dr. Daniel will not be surprised at the brilliance of this new production. As one dips deeper into "Dr. Courtenay's" narrative the feeling grows that this man had real being, and by the time Dr. Bruno appears we know that we have been taken behind the scenes of an individual life—that we are witnessing a dissection of the mind and soul of a wonderfully gifted but most intensely human fellow creature.

Dr. Bruno is a man we have all met—or would like to meet—and the peculiar ideas he advances to his friend Dr. Courtenay

*From The American Journal of Clinical Medicine.

might easily be heard after the cigars are lit in the den of any well-read modern physiologist. But his tangled and tragic life-story, which is so graphically told, could belong to but few men—and fewer men still could tell it.

Dr. Bruno, after suggesting to his friend Dr. Courtenay, who tells the story, the desirability of "devoting to science the condemned criminal, say the negro rapist who casts his shadow over our fair land more baleful than the lethean shade of the deadly upas—not to *dissection* as the people understand it, but to experimental study on the internal organs to solve the problems of immunity, fermentation and glandular action," experiments himself with a drug which causes a suspension of the vital processes—enables man to assume the condition of the hibernating animal.

Disgusted with a society which clumsily destroys the useless criminal while refusing the use of his live carcass to science Dr. Bruno determines to take "his own medicine," and does so, leaving a note behind, detailing in precise terms just what will occur and what must be done when he begins to return to life. He has "timed" the dose to act for six months and assures his friend Courtenay that at the end of that time he will awaken.

In case of accident he leaves a manuscript containing the story of his life—the weirdest, most tragic, most pitiful story man could tell. He had married, as he supposed, his own sister; loses his identity—and his wife—and devotes himself, upon regaining his reason, to a minute study of the phenomena of *sleep*. His present experiment is the final test of the correctness of the theories he has elaborated.

Dr. Bruno *does* awake after six months of death-like sleep—only to die in the arms of his daughter, who was born after her mother had fled in horror from home and husband upon being told the secret of her birth by her supposed father.

If ever material for a plot existed in abundance it exists here and the reader of "The Strange Case of Dr. Bruno" will own, when he puts down the book, that Dr. Daniel has wrought marvellously well with the matter at his disposal.

Beyond the interest of the story there remains the insistent thought, *are* Dr. Bruno's ideas so preposterous after all? We can suspend life for a certain time, why not for longer? And, if to find out our limitation in this direction it is necessary to have human subjects to experiment upon, why should those subjects not be selected from such specimens of humanity as have preyed in the most obnoxious manner upon their fellows? The law exacts life, why should that life not be taken in a way which will prove useful to humanity at large instead of bunglingly at the end of a rope? The condemned man would be carefully fed and tended; he would serve his purpose and finally would succumb to a hypodermatic injection of some instantly fatal poison or—perchance—he would be (later on) put to sleep for an indefinite term to awake at last a new man under new conditions.

The reading of Dr. Bruno will at least make a man think strange thoughts and realize that many things may be which are not.—*George H. Candler, M. D., Chicago, Ill.*

